# Building neurocritical care education in Brazil: from recognition to action

**DOI:** 10.1055/s-0046-1822636

**Published:** 2026-06-25

**Authors:** Álvaro Réa-Neto, Caroline Uliana Rossi, Jeane Cristina Fonseca Vieira

**Affiliations:** 1 Centro de Estudos e de Pesquisas em Terapia Intensiva, Curitiba PR, Brazil.; 2 Universidade Federal do Paraná, Programa de Pós-Graduação em Medicina Interna e Ciências da Saúde, Curitiba PR, Brazil.; 3 Instituto de Neurologia de Curitiba, Curitiba PR, Brazil.

Standardized neurocritical medical care education in Brazil has evolved from an academic concern to an urgent public health priority. Persistent disparities in care quality demand immediate curricular reforms as both a clinical necessity and a strategic investment in health care equity.

In this context, the landmark multicenter study by Marazzi and colleagues 1 (2025) in this issue provides Brazil's first assessment of neurocritical training gaps. Their findings reveal that only half of neurologists and intensivists receive formal neurocritical care education during residency, and even trained professionals show deficits in advanced monitoring and procedural skills. These quantified deficits provide an evidence-based foundation for curricular reform, shifting the discourse from problem identification to solution implementation.

Notably, the Neurocritical Brazil Study Group has demonstrated that neurologically ill patients account for approximately 10% of ICU admissions nationwide, with mortality rates 1.7 times higher than non-neurocritical patients. 2 This crisis worsens when considering Brazil's acute specialist shortage, where only 78 physicians dual-certified neurologist-intensivists serve over 200 million people. 3 This disparity reveals a systemic failure that compromises timely, specialized care for critically ill neurological patients across the country.

These systemic challenges are further exacerbated by alarming gaps between clinical training and practitioner confidence. While 95% receive stroke and subarachnoid hemorrhage (SAH) management, only 70% reported confidence in independently managing SAH cases, dropping to 61% for traumatic brain injury. Most concerning, confidence levels fall below 60% for fundamental neurocritical interventions including post-cardiac arrest care, refractory intracranial hypertension, and spinal cord injury management. This discrepancy suggests deficiencies in both training accessibility and educational quality, as residents complete rotations without developing the procedural competence or clinical autonomy required in advanced neurocritical practice.

Particularly notable is the limited exposure to essential neuromonitoring modalities. Only 24% of trainees gain experience with multimodal monitoring, 23% with transcranial Doppler, and 21% with electroencephalogram (EEG) interpretation. These tools set modern neurocritical care apart from general intensive care. This deficit extends beyond resource concentration in tertiary centers, revealing a predominantly observational training model. The minimal inclusion of simulation-based education reaches just 6.6% of trainees. This 6.6% adoption rate contrasts sharply with the 85-90% 4 utilization in leading international neurocritical programs, representing a critical gap in Brazil's preparation of clinicians for high-acuity scenarios where simulation serves as the global cornerstone for skill acquisition and crisis preparedness. 4 International benchmarks provide valuable insights. The United States experience demonstrates how formal recognition and standardized curricula can transform a discipline. 5 Since the United Council for Neurologic Subspecialties began accrediting programs in 2007, neurocritical care fellowships grew from fewer than 20 to nearly 70, producing hundreds of board-certified specialists annually. Similarly, Europe approaches emphasize competency frameworks and interprofessional collaboration. Both models recognize neurocritical care as a distinct specialty requiring dedicated training, rather than simply combining neurology and intensive care competencies. 6

Contemporary neurocritical care calls for more than monitoring intracranial pressure. Physicians need individualized treatment skills, know when to use or skip advanced monitoring, use noninvasive techniques highlighted in recent guidelines, discuss interventions with neurosurgeons, and understand expected outcomes after acute brain injuries. These practices require special training, a team approach, and cost analysis. 7 8

Brazil should adapt, not copy, international models. Three key priorities arise: developing a national consensus curriculum for neurology and intensive care residents; establishing formal fellowships at neurocritical care centers to serve as training hubs; and collaborating with regulatory authorities to formally recognize neurocritical care as a speciality, thereby setting standards and providing incentives for training. Success depends on a united effort from academic institutions and professional societies, particularly AMIB, ABN and ABNI. Current disparities in training quality reflect systemic gaps that demand real cooperation, not fragmented initiatives. Sustainable change requires institutional commitment and collaborative frameworks to set consistent standards for neurocritical education nationwide. Pragmatic implementation can accelerate progress. While not every city requires a fellowship program, all residency training must incorporate core competencies: basic neuromonitoring, intracranial pressure management, and neuroprognostic decisions-making. Cost-effective strategies include structured clinical rotations, tele-education platforms, regional simulation centers, and faculty development programs. The Marazzi et al. study revealed that more than half of respondents expressed interest in specializing in neurocritical care, indicating significant untapped potential among neurologists and intensivists. Although these professionals recognize both the challenges and rewards of the field, they currently lack adequate structured training pathways and mentorship opportunities.

Geographic gaps make the challenge harder. The survey's Southeast focus offers insight, but likely understates national problems by omitting resource-poor regions. Even top centers in São Paulo and Rio de Janeiro have fragmented training, which suggests worse conditions elsewhere. This gap is both educational and a matter of equity, as all patients should get equal care. Specialized neurocritical units greatly improve outcomes, making this an ethical and clinical priority. 9 10 Solutions include competency-based curricula, regional training centers, subspecialty recognition, and investing in educational infrastructure.

Brazil's medical community must turn this urgent need into action. Meanwhile, neurocritical patients pay the price for delay: every day without standardized training keeps avoidable deaths high. We must move from awareness to collective action and create a strong, high-quality national framework for neurocritical education.
